# Syncope in the Emergency Department

**DOI:** 10.3389/fcvm.2019.00180

**Published:** 2019-12-03

**Authors:** Roopinder K. Sandhu, Robert S. Sheldon

**Affiliations:** ^1^Division of Cardiology, University of Alberta, Edmonton, AB, Canada; ^2^Department of Cardiac Sciences, Libin Cardiovascular Institute of Alberta, University of Calgary, Calgary, AB, Canada

**Keywords:** syncope, emergency department (ED), initial evaluation, risk stratification, outcomes

## Abstract

Syncope is a common presentation to Emergency Departments (EDs). Estimates on the frequency of visits (0.6–1.7%) and subsequent rates of hospitalizations (12–85%) vary according to country. The initial ED evaluation for syncope consists of a detailed history, physical examination and 12-lead electrocardiogram (ECG). The use of additional diagnostic testing and specialist evaluation should be based on this initial evaluation rather than an unstructured approach of broad-based testing. Risk stratification performed in the ED is important for estimating prognosis, triage decisions and to establish urgency of any further work-up. The primary approach to risk stratification focuses on identifying high- and low-risk predictors. The use of prediction tools may be used to aid in physician decision-making; however, they have not performed better than the clinical judgment of emergency room physicians. Following risk stratification, decision for hospitalization should be based on the seriousness of the underlying cause for syncope or based on high-risk features, or the severity of co-morbidities. For those deemed intermediate risk, access to specialist assessment and related testing may occur in a syncope unit in the emergency department, as an outpatient, or in a less formal care pathway and is highly dependent on the local healthcare system. For syncope patients presenting to the ED, ~0.8% die and 10.3% suffer a non-fatal severe outcome within 30 days.

## Introduction

Syncope is a frequent reason for Emergency Department (ED) visits. Although estimates are largely influenced by studies reflective of specific populations, the accuracy of data collection and the definition of syncope used, numbers range from 0.6 to 1.0% in North America and 0.9–1.7% in Europe (1–3). The rates of subsequent admission to the hospital from the ED also vary depending on country, ranging from 12 to 15% in Canada (4, 5), 31–38% in Italy (6, 7), 49% in the United Kingdom (8), and 46–86% in the United States of America (9, 10).

Syncope is a symptom that may be the final presentation for a variety of conditions ranging from benign to life threatening. The most common causes of syncope seen in the ED are due to reflex syncope (35–48%) followed by orthostatic hypotension (4–24%), cardiac (5–21%), non-syncope transient loss of consciousness (TLOC) causes and anywhere from 17 to 33% of syncope presentations may remain unexplained (11–15).

## Initial ED Evaluation

An initial assessment in the ED involves a detailed history, physical exam (including standardized orthostatic vitals defined as blood pressure and heart rate changes in lying and sitting positions, on immediate standing and after 3 min of upright posture) and a 12-lead electrocardiogram (ECG) to determine whether an underlying cause of syncope can be identified and to help with prognostication (16, 17). Further diagnostic testing including blood work, cardiac and neurological tests and specialist evaluation should be mainly driven by the initial evaluation and a differential diagnosis that makes the extent and context of an additional work-up appropriate.

## Risk Stratification

The role of risk stratification that occurs during the ED evaluation is important for several reasons: (i) it helps to estimate prognosis, (ii) influences triage decisions, (iii) establishes urgency for additional tests and specialist evaluation, and (iv) ensures appropriate discussions occur with patients. Yet, no optimal approach to risk stratification exists and as a result different approaches are being utilized.

Numerous prediction tools exist to help reduce unnecessary hospitalizations and healthcare costs related to syncope care. Examples of risk stratification tools evaluated in prospective studies are shown in Table 1. However, these scores have not been widely adopted into clinical practice because of important limitations including inconsistent definitions of syncope, outcomes, outcome time frames, and predictors, inclusion of “obvious” serious causes, small sample size, and limited external validation. To try and address some of those limitations, an individual patient data meta-analysis was performed to externally validate the available syncope prediction tools and compare them with clinical judgment (23). Syncope risk stratification tools did not show better diagnostic yield or prognostic yield in predicting serious short-term outcomes compared with clinical judgment. This study used older risk scores. A new syncope risk score was recently developed, the Canadian Syncope Risk Score (CSRS), which incorporates clinical factors, ECG and elevated troponin (>99th percentile of normal population) and assumed diagnosis in the ED (22). The CSRS performed better when comparing area under the curve (AUC) than not only cardiac biomarkers at predicting death and adverse outcomes but also cardiac biomarkers combined with older risk scores (24). The work underlying the scores do consistently identify certain predictors from the history, physical exam and ECG that are associated with a worse prognosis at 1–2 years follow up. Identifying those factors as either high-risk (suggesting serious condition) or low-risk (suggesting benign condition) has also been used for risk stratification (17). Precise definitions for high-, intermediate-, and low-risk patients evaluated in the ED after a syncope event do not exist. Available data makes this challenging because of variability in risk markers, study endpoints and adverse event rates among studies. An alternative to this approach has been to use risk markers from history, physical exam, laboratory investigations, and ECG to divide patients according to short-term risk (adverse outcome in the ED or post-30 days after ED discharge) and long-term risk (up to 1-year) (16).

**Table 1:** Example of syncope risk scores evaluated in prospective studies.

**Study/year**	**Cohort (*N*)**	**Risk factors**	**Score**	**Endpoint**	**Results**
Martin et al. (18)	252	Age > 45 years Abnormal EKG Ventricular arrhythmia Heart failure	0–4 (1 point each item)	1-year severe arrhythmias or arrhythmic death	0% score 0
Colivicchi et al. (19)	270	Age > 65 years Abnormal EKG Cardiovascular disease Lack of prodrome	0–4 (1 point each item)	1-year mortality	0% score 0 0.6% score 1 14% score 2 29% score 3 53% score 4
Quinn et al. (20)	684	Abnormal EKG Heart failure Shortness of breath Hematocrit < 30% SBP < 90 mmHg	No risk: 0 items Risk: ≥ 1 item	Serious events at 7 days	98% sensitive, 56% specificity
Brignole (21)	260	Palpitations (+4) Abnormal EKG/CVD (+3) Syncope effort (+3) Syncope supine (+2) Autonomic prodrome (−1) Predisposing factors (−1)	Sum of + and – points	2-year mortality Cardiac syncope probability	2% score < 3 21% score ≥ 3 2% score < 3 13% score 3 33% score 4 77% score >4
Reed et al. (8)	550	BNP ≥ 300 pg/mL HR ≤ 50; q waves EKG Fecal occult blood Hemoglobin ≤ 90 g/L Chest pain with syncope O_2_ ≤ 94% room air	No risk: 0 items Risk: ≥ 1 item	1-month serious events or death (occurred in 7.1%)	87% sensitive, 65% specificity, 98% negative predictive value
Thiruganasambandamoorthy et al. (22)	4,030	Predisposition VVS symptoms (−1) History of heart disease (+1) SBP <90 or >180 mmHg (+2) Elevated troponin (+2) QRS axis < −30″ or >100″ (+1) QRS duration > 130 ms (+1) QTc interval > 480 ms (+2) Diagnosis of VVS in ED (−2) Diagnosis of cardiac syncope in ED (+2)	Add the + and – points (from −3 to 11)	Serious events at 30 days	0.4–0.7% score −2 to −3 1.2–1.9% score 0 to −1 3.1–8.1% score 1–3 12.9–19.7% score 4–5 28.9–83.6% score 6–11

## Disposition From ED

Following risk stratification, a decision regarding disposition must occur.

The decision for hospitalization is primarily based on the seriousness of the identified diagnosis or based on high-risk features identified during the initial evaluation. There is no strong evidence that hospitalization improves outcomes and in patients without a serious condition (e.g., reflex syncope or low-risk features) hospitalization has not been shown to improve short- and long-term outcomes and therefore these patients should be managed in an outpatient setting. The main role of hospitalization should be to expedite treatment or further diagnostic work-up (9, 25).

**Figure 1:**
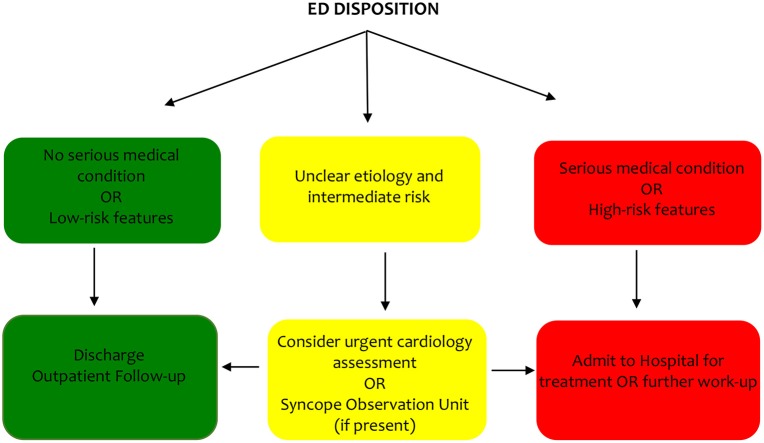
Proposed disposition strategy from ED.

The optimal triage strategy for the “intermediate” risk patient remains a challenge. One proposed strategy is the syncope unit, aimed at reducing rates of under/misdiagnosis, hospital admission and costs (26). The key to a syncope unit is having advanced access to specialist assessment and related testing using an evidence-based approach. The unit may be located in the inpatient (cardiology or internal medicine department or ED) or outpatient setting (i.e., Rapid Access Blackout Clinic or Faint/Fall clinic) with referrals coming from the ED or community practitioners/cardiologists, depending on the location and the interaction can occur with an in-person or web-based evaluation. There are only two small, randomized clinical trials that have evaluated ED-based syncope units compared with usual care (27, 28). The results demonstrate higher diagnostic yield, lower hospital admission, reduced costs and no increase in adverse outcomes in patients randomized to the syncope unit. The ability to integrate a syncope unit is highly dependent on the structure and funding of an individual healthcare system and may not be required universally. A proposed strategy for disposition for the ED taking into consideration different healthcare systems is shown in Figure 1.

The European Heart Rhythm Association task force (26) developed preliminary quality indicators, based on consensus, for evaluation of a syncope unit and includes:

- An absolute rate of undiagnosed TLOC should be reduced by 20%.
- <20% of low-/intermediate-risk TLOC patients should be admitted from the ED.
- The syncope unit should have a 20% reduction in costs relative to usual practice and improved outcomes (i.e., <5% readmissions for syncope and <20% of paced patients with recurrence at 1-year).

For example, in Canada, two of these quality indicators (<20% low-/intermediate-risk TLOC patients should be admitted from the ED and <5% readmissions for syncope at 1-year) have been met without introduction of a syncope unit (4, 5, 29). More studies are needed to assess the clinical and economic effectiveness of these different approaches compared to usual care.

## Guideline Comparison for ED Evaluation of Syncope

The initial syncope evaluation of a detailed history, physical exam (including orthostatic vitals) and 12-lead ECG is a class I recommendation in the 2017 American College of Cardiology/American Heart Association/Heart Rhythm Society (ACC/AHA/HRS) guidelines while the European Society of Cardiology (ESC) document gives no class recommendation.

A key role to perform in the ED evaluation of syncope is risk stratification. When the underlying cause of syncope has been identified in the ED then risk stratification is more apparent. However, when the diagnosis is not clear then several approaches have been proposed for risk stratification including identifying risk factors with or without categorizing patients as low-, intermediate-, or high-risk, risk stratification tools or clinical judgment. Both guidelines give a class IIb recommendation (weak evidence) for use of ED prediction tools. One of the most marked distinctions between the guidelines is disposition from the ED for patients deemed “intermediate” risk. The ESC guidelines provide a strong recommendation (class I) for an ED or outpatient syncope unit evaluation instead of admission to the hospital for this subgroup. While the ACC/AHA/HRS guidelines suggest use of a structured ED observation (class IIa) can be an effective strategy. Both recommendations are based on the same limited studies.

## Outcomes

Among studies that have evaluated short-term (7–30 day) outcomes of patients presenting to the ED with syncope, the composite estimate for death was 0.8% and 10.3% had suffered a non-fatal severe outcome (significant new diagnosis, a clinical deterioration, serious injury with recurrence, or a significant therapeutic intervention) (17). Approximately 6.9% had a non-fatal severe outcome while in the ED and another 3.6% of syncope patients after ED discharge. In a meta-analysis of consecutive patients presenting to the ED, pooled estimates for mortality at 1-year was 8.4% (95% CI 6.7–10.2%), 8.9% (95% CI 7.4–10.6%) at 1.5 years, and 11.0% (95% CI 7.0–16.8%) at 2-years (30). In addition to high heterogeneity or few studies, many of these observational studies included patients both discharged or admitted from the ED. An Italian study (31) evaluating mortality based on disposition found 1.8% of syncope patients who were discharged from the ED died compared to 14.7% who were admitted. Almost half of admitted patients were 65 years or older and had significantly higher burden of cardiovascular comorbidity compared to those patients discharged from the ED. A study from Canada demonstrated both short- and long-term mortality rates among syncope patients discharged from the ED were very low (30 day 0.4% and 1-year 3.0%) (5). Among admitted patients, mortality rates were at least four times higher at 30 day and at least three times higher at 1-year among admitted patients compared to those who were discharged.

## Healthcare Utilization

There are few data that report on the costs of syncope care exclusively for syncope patients in the ED. A study that examined costs of patients with syncope admitted and discharged from the ED found of the total costs (530 million CDN) over a 6-year period, the highest proportion was attributed to patients discharged from the ED (317 million CDN) because this cohort represented 85% of the study population (5). The highest proportion of annual costs were due to hospitalizations for each of the cohorts (admitted/discharged with syncope, admitted/discharged with an alternative diagnosis, discharged from the ED); however, for syncope patients who were discharged home from the ED, outpatient plus physician claims costs equaled those of hospitalization costs.

## Author Contributions

RKS wrote the content of the manuscript. RSS provided critical review.

### Conflict of Interest

The authors declare that the research was conducted in the absence of any commercial or financial relationships that could be construed as a potential conflict of interest. The reviewer RF declared a past co-authorship with one of the authors RSS to the handling editor.
